# Anti-Blooming Clocking for Time-Delay Integration CCDs

**DOI:** 10.3390/s22197520

**Published:** 2022-10-04

**Authors:** Denis Szymon Piechaczek, Olaf Schrey, Manuel Ligges, Bedrich Hosticka, Rainer Kokozinski

**Affiliations:** 1 Fraunhofer Institute for Microelectronic Circuits and Systems, Finkenstr. 61, 47057 Duisburg, Germany; 2 Department of Electronic Components and Circuits, University of Duisburg-Essen, Bismarckstr. 81, 47057 Duisburg, Germany

**Keywords:** time delay integration, charge-coupled device, responsivity, photon transfer curve, conversion gain, signal-to-noise ratio, blooming

## Abstract

This paper presents an investigation of the responsivity of a time-delay integration (TDI) charge-coupled device that employs anti-blooming clocking and uses a varying number of TDI stages. The influence of charge blooming caused by unused TDI stages in a TDI deployed selection scheme is shown experimentally, and an anti-blooming clocking mechanism is analyzed. The impact of blooming on sensor characteristics, such as the responsivity, the conversion gain, and the signal-to-noise ratio, is investigated. A comparison of the measurements with and without this anti-blooming clocking mechanism is presented and discussed in detail.

## 1. Introduction

**Figure 1:**
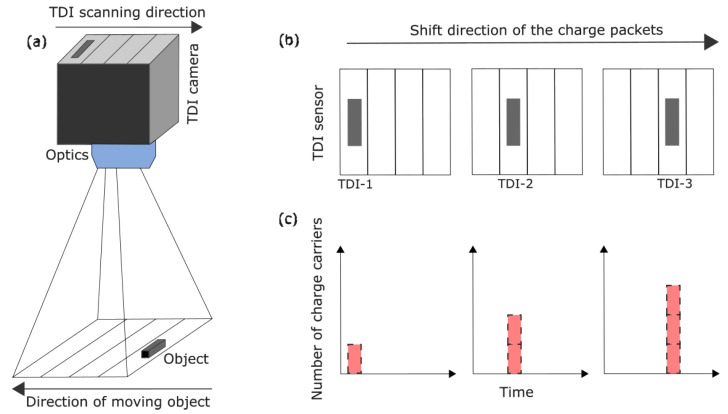
Working principle of the time-delay integration process. (**a**) TDI camera detects a moving object. (**b**) Position of the moving charge packet in a pixel line that is synchronous with the object’s movement. (**c**) Charge accumulation over time as a result of the TDI mode.

Modern remote sensing systems require low-light-level detection capabilities, which can be provided by charge-coupled devices (CCDs) operated in time-delay integration (TDI) mode in applications, where either sensor or the target are moving at constant velocity [1]. Time-delay integration is a special line scanning mode in which a moving scene is integrated multiple times over a number of pixel lines, called TDI stages. For this, the transfer of the photo-generated charge packet is synchronized with the relative movement of this scene. Hence, due to the cumulative exposure of the sensor, the effective exposure time increases linearly with the number of active TDI stages, resulting in an increased number of charge carriers within the pixels [2]. This principle is sketched in Figure 1.

Consequently, TDI-CCDs show greatly enhanced responsivities to allow faster operations in terms of line rates, which offers advantages in particular for earth observation applications [3]. Moreover, since the illumination of the sensor can vary for different cases, a TDI sensor usually has the capability to select between a particular number of active TDI stages. This allows the adjustment and variation of the effective exposure time so that a bright scene uses a lower number of TDI stages, whereas a higher number of TDI stages is required for a darker scene. Due to these varying adjustments, an optimum between exposure time, saturation level, and signal charge transfer are achieved. Here, the saturation level denotes the maximum amount of charge carriers that a pixel can store and transfer without sacrificing performance. When the charge in the pixel exceeds this limit, charge carriers start to overflow into adjacent pixels, which is called blooming. Hence, an unmatched effective exposure time can cause blooming, especially if no anti-blooming structure/mechanism is integrated at all. As a result, the image quality will be degraded significantly [4].

Due to the use of a reduced number of TDI stages, an accumulation of charge carriers in unused stages can also result in an overflow into the active TDI region. Therefore, removing the photo-generated charge carriers from the unused region is necessary to prevent blooming. This can be done either by dumping the charge in a vertical [5], or lateral [4] anti-blooming drain or by shifting them in the opposite direction into a sink. The latter anti-blooming technique was already proposed in [6] and [7]. Still, an actual examination of the operation of this anti-blooming technique, as well as a comparison of the sensor operated in different TDI modes with and without the anti-blooming technique, has not been presented up to now. Therefore, this paper discusses the anti-blooming technique by shifting in the opposite direction and provides a detailed analysis of the effect on specific device parameters used for sensor characterization.

For this, we will first present the design of the used TDI CCD sensor and the readout circuitry, as well as the implemented timing schemes. Afterward, measurements of the responsivity for different numbers of active TDI stages will be compared, and the effect of the inactive region on these measurements will be discussed. The conversion gain and the signal-to-noise ratio, which are crucial for sensor characterization, will be considered for these different circumstances, and their reliability will be assessed for the use case of TDI CCD sensors. Finally, a discussion and conclusions of the measurements will be presented in detail.

## 2. Design of the TDI CCD Chip

**Figure 2:**
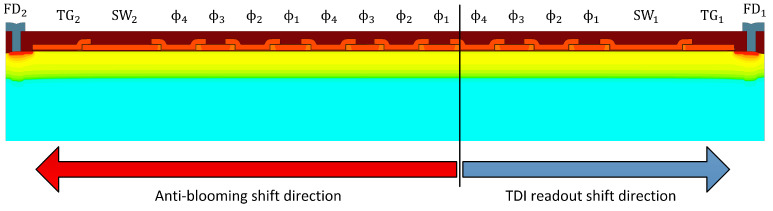
Cross-section of the TDI-CCD. The black line divides the anti-blooming shift region and the TDI shift region.

**Figure 3:**
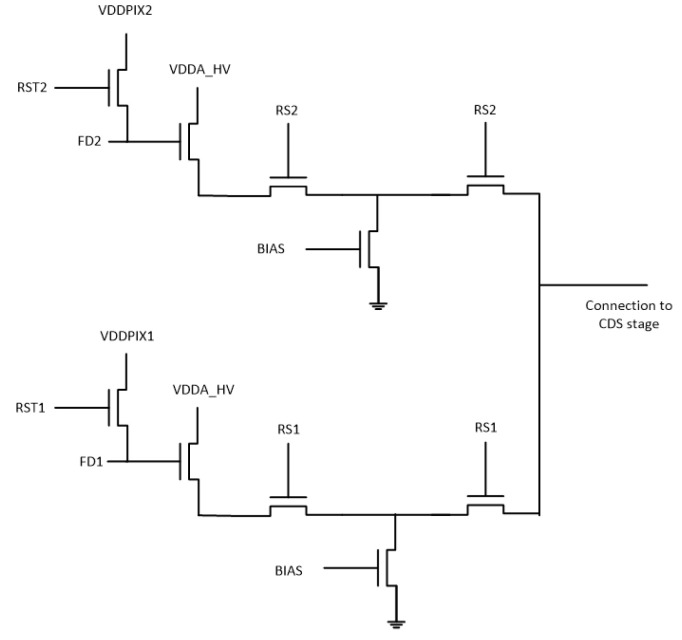
Design of the double source follower circuitry.

The employed TDI CCD sensor was developed by Fraunhofer IMS [8]. The chip consists of a pixel matrix having 320 columns, each with a length of 128 TDI stages, and offers the possibility to change the number of active TDI stages in steps 1, 32, 64, and 128. In addition, because of the sensor’s design, a shifting of charges in both directions is possible. Therefore, a summing well (SW), a transfer gate (TG), a floating diffusion (FD), and a source follower circuitry are available on both sides. The cross-section depicted in Figure 2 shows a divided pixel column where one section serves as an anti-blooming structure while the other section is used for the readout of the signal. In the latter case, the signal transferred in the TDI readout direction is shifted from pixel to pixel until it reaches the summing well, where it is collected and transferred via the transfer gate to the sense node. Furthermore, each TDI column possesses a source follower circuitry at both sides, which is connected in front of the correlated double sampling stage (CDS stage), as seen in Figure 3. To evaluate the signal, it is first processed by the CDS stage and then stored in a sample-and-hold (S&H) circuitry. The digitization is carried out finally by a comparator and an externally provided Gray code and linear ramp.

**Figure 4:**
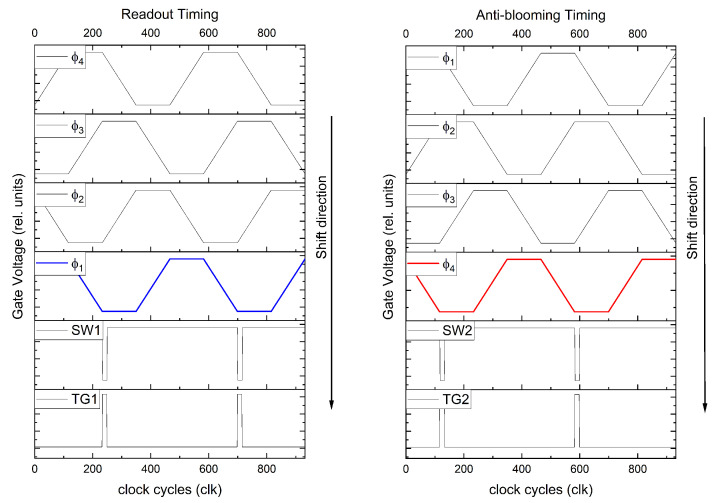
Timing scheme for TDI readout mode (**left**) and for anti-blooming mode (**right**).

The timing schemes used for bidirectional shifting are shown in Figure 4. As can be seen in the left part of the figure, the shift direction for the readout mode is $\Phi_{4}\to \Phi_{3}\to \Phi_{2}\to \Phi_{1}\to {\mathrm{SW}}_{1}\to {\mathrm{TG}}_{1}\to {\mathrm{FD}}_{1}$. When comparing it with the right-hand side of the figure, it can be seen that both timing schemes shift in opposite directions. Thus, the anti-blooming timing is $\Phi_{1}\to \Phi_{2}\to \Phi_{3}\to \Phi_{4}\to {\mathrm{SW}}_{2}\to {\mathrm{TG}}_{2}\to {\mathrm{FD}}_{2}$. Since only one side of the TDI column is read out, the signals that reach the FD${}_{2}$ will be removed by RST${}_{2}$ and VDDPIX${}_{2}$. Therefore, the row select RS${}_{2}$ is operated in continuous blocking mode, while RS${}_{1}$ passes the signal to the floating diffusion FD${}_{1}$ and the CDS stage.

## 3. Experimental Results

The characterization of the TDI chip, fabricated at Fraunhofer IMS, was performed in a dark chamber. An illumination source that provides monochromatic light of the wavelength λ=850nm, at which a high quantum efficiency is obtained for this sensor, was operated in continuous mode. The irradiance *E* was increased in $25\;{µW/\mathrm{cm}}^{-2}$ steps after each measurement until the highest adjustable irradiance $E_{\max}=350\;{µW/\mathrm{cm}}^{-2}$ was reached. This measurement was repeated for different numbers of TDI stages. The evaluation was carried out according to the EMVA Standard 1288 Release 3.1 [9].

### 3.1. Responsivity

The exposure of the sensor causes the generation of charge carriers, which scales linearly with the irradiance as long as the amount of charge carriers does not exceed the saturation level. This input–output relation is defined as responsivity. To verify the linearity, the mean Gray values $\mu_{y}$ given in digital numbers DN are depicted in relation to the number of photons $\mu_{p}$ where the slope yields the responsivity *R* in accordance with

(1) $$\begin{array}{c} R=\frac{\mu_{y}-\mu_{y.\mathrm{dark}}}{\mu_{p}}. \end{array}$$

Here, $\mu_{y.\mathrm{dark}}$ denotes the dark offset that results from measurements without irradiance. The total number of photons that hit a pixel area *A* during the exposure time $t_{\exp}$ after the $N_{\mathrm{TDI}}$ stages is obtained by the formula

(2) $$\begin{array}{c} \mu_{p}=\frac{AEt_{\exp}\lambda N_{\mathrm{TDI}}}{hc}, \end{array}$$

where *h* is Planck’s constant and *c* is the speed of light.

**Figure 5:**
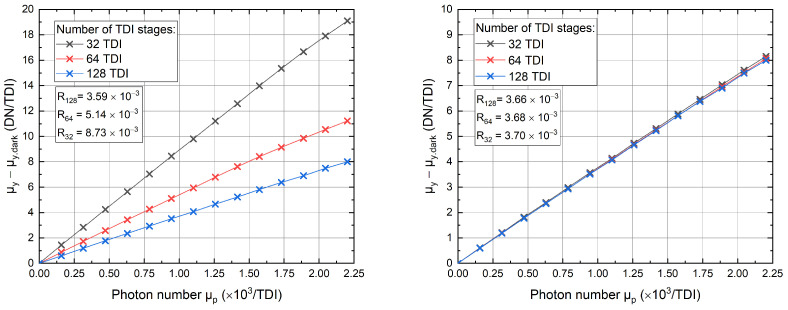
Responsivity curves measured without (**on the left**) and with (**on the right**) integration of the anti-blooming clocking for different numbers of TDI stages.

Since the photon number and mean Gray values increase linearly with the number of TDI stages, the responsivity should result in a constant and equal value for each TDI selection. To verify this, the responsivity curves for different numbers of TDI stages were measured. At first, the unused stages were set inactive, meaning that there was no charge shift present in this region, since all TDI gates were set to low. Afterward, an anti-blooming mechanism was implemented to include a shifting of unused TDI stages in the opposite direction to remove the charge generated in this area as soon as it reaches the second floating diffusion. The measured responsivity curves are shown in Figure 5. To better compare these different TDI modes, the mean gray values and the photon numbers have been normalized to one single TDI stage by dividing by $N_{\mathrm{TDI}}$.

As a result, for the measurement conducted without an anti-blooming mechanism, we obtain responsivities that differ in dependence on the used TDI stage, whereas the responsivities with implemented anti-blooming yield equal values. The reason is the occurrence of blooming caused by the accumulation of charge carriers in regions where the TDI stages are inactive since they cannot be removed. Thus, the accumulated charge carriers start to flow into the active region as the number of charge carriers exceeds the saturation level of the pixel. Here the saturation is reached at around 1000 DN or equivalently 160 ke${}^{-}$. Moreover, due to the increased number of charge carriers caused by blooming, the charge packet will approach the Si-SiO${}_{2}$ interface [10] resulting in a degradation of the charge transfer efficiency [11]. This can be observed by comparing the linearities of both measurements in Figure 5, which are worse for the measurement without anti-blooming.

**Figure 6:**
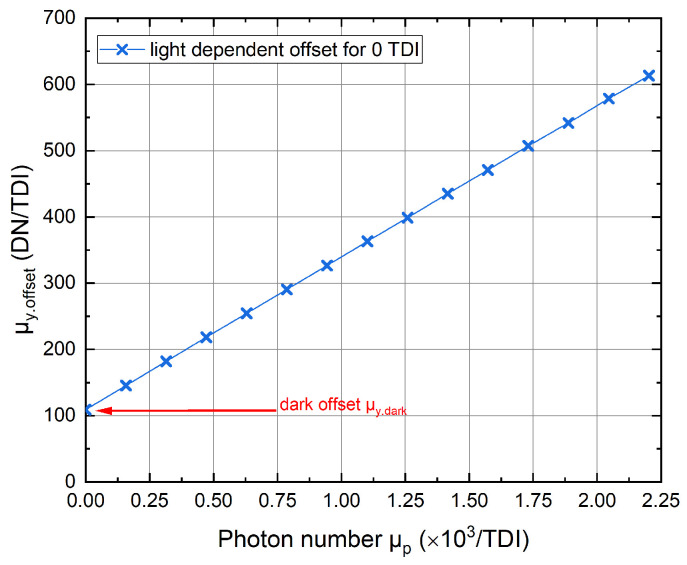
Signal $\mu_{y.\mathrm{offset}}$ in dependence on the photon number. Red arrow marks the dark offset $\mu_{y.\mathrm{dark}}$ for $E=0\;µW/{\mathrm{cm}}^{2}$.

Consequently, because the generation of charge carriers in the inactive region also depends on the irradiance, we obtain a light-dependent offset in the measurements if no anti-blooming mechanism is implemented. To show that this offset behaves linearly and corresponds to the deviation identified in Figure 5 we adjusted all TDI stages inactive, while the summing well, the transfer gate, and the readout circuitry operated as described in Section 2. The light-dependent offset, which increases steadily with the photon number, is depicted in Figure 6. However, since this curve is obtained for 128 deactivated TDI stages, we need to calculate the proper contribution of the offset for a different number of deactivated TDI stages. The corrected mean Gray values result from

(3) $$\begin{array}{c} \mu_{\mathrm{corr}}=\mu_{y}-\frac{\mu_{y.\mathrm{offset}}-\mu_{y.\mathrm{dark}}}{N_{\mathrm{TDI}.\mathrm{active}}}\cdot \frac{128-N_{\mathrm{TDI}.\mathrm{active}}}{128}, \end{array}$$

where the normalized offset is multiplied by the relative number of deactivated TDI stages.

**Figure 7:**
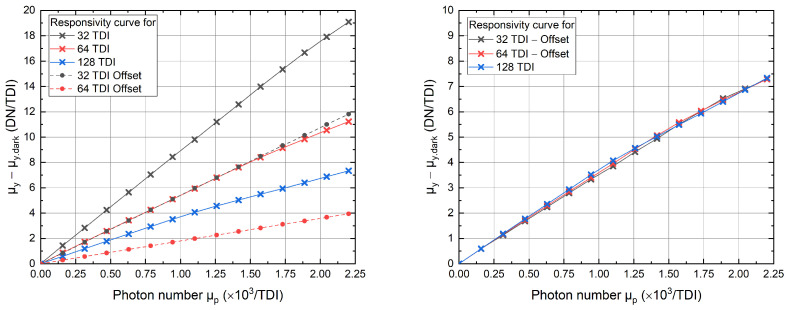
Responsivity curves measured without integration of the anti-blooming clocking for different numbers of TDI stages. Dotted curves in the figure on the left show the light-dependent offset for 32 and 64 TDI stages. The plot on the right shows responsivity curves after the offset has been subtracted.

For better comparison, we have plotted the responsivity curves without an anti-blooming mechanism and the calculated offset curves for 32- and 64 TDI on the left-hand side of Figure 7. The subtraction of the offset yields identical responsivity curves for different TDI modes, as seen in the right diagram. This proves the initial statement that if no anti-blooming mechanism is implemented, the charge carriers, which are generated in the inactive region, start to flow into the active region, contributing to the signal. As already shown, the cancellation of this light-dependent offset takes place by using an anti-blooming implementation which avoids the accumulation of charge carriers in the inactive region of the CCD matrix.

### 3.2. Photon Transfer Method and Signal-to-Noise Ratio

In the previous section, we have shown the occurrence of a light-dependent offset if no proper anti-blooming mechanism is implemented and how the responsivity can be used to identify this effect. This finding indicated that the measurement of the responsivity with different numbers of TDI stages is a crucial quantity for verifying the reliability of a TDI sensor. For this reason, the influence of blooming on the conversion gain and the signal-to-noise ratio has been investigated since both quantities play an essential role in characterizing image sensors.

#### 3.2.1. Photon Transfer Curve

To examine the influence of blooming on the conversion gain, which is the conversion factor of electrons to digital numbers, we used the photon transfer method [12]. The photon transfer method depicts the measured photo-induced variance $\sigma_{y}^{2}$ versus the mean Gray values $\mu_{y}$, where the variance is given by [13]

(4) $$\begin{array}{c} \sigma_{y}^{2}=K^{2}\left(\sigma_{d}^{2}+\sigma_{e}^{2}\right)+\sigma_{q}^{2} \end{array}$$

and depends on different noise sources: The quantization noise $\sigma_{q}^{2}=1/12\;{\mathrm{DN}}^{2}$ as well as the sensor readout and the amplifier circuit-related distributed noise $\sigma_{d}^{2}$ are signal independent, while the shot noise contribution $\sigma_{e}^{2}$ is equal to the number of accumulated electrons $\sigma_{e}^{2}=\mu_{e}$. Using Equation (4) and the relation $\mu_{y}=\mu_{y.\mathrm{dark}}+K\mu_{e}$ yields the variance of the measured signal [14]

(5) $$\begin{array}{c} \sigma_{y}^{2}=\sigma_{y.\mathrm{dark}}^{2}+K\left(\mu_{y}-\mu_{y.\mathrm{dark}}\right) \end{array}$$

where the slope of the photon transfer curve corresponds to the conversion gain *K* and $\sigma_{y.\mathrm{dark}}^{2}=K^{2}\sigma_{d}^{2}+\sigma_{q}^{2}$ denotes the dark noise variance.

**Figure 8:**
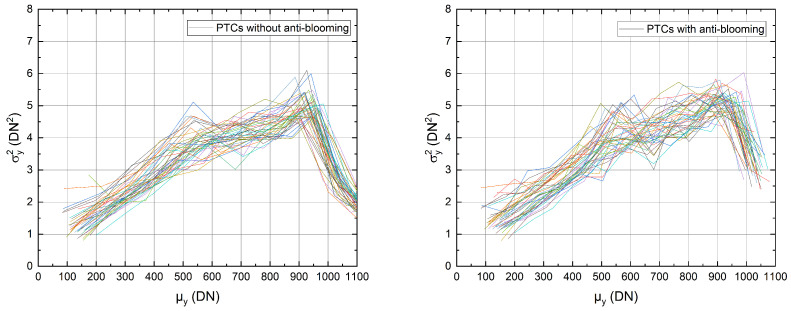
PTCs without implemented anti-blooming clocking (**left**) and PTCs with implemented anti-blooming clocking (**right**) for 64 TDI stages.

The following measurement was evaluated for 64 TDI stages and 40-pixel columns with and without anti-blooming clocking at a line rate of 16.7kHz. Figure 8 shows the resulting photon transfer curves.

As can be seen, both diagrams exhibit a kink at 600DN, most probably caused by surface contact of the charge packets, which distorts the evaluation of the conversion gain. Therefore, the evaluation concerns the interval 100DN–500DN. As a result, we obtain the mean conversion gains:

(6) $$\begin{array}{cc} K_{\mathrm{noAB}} & =\left(6.30\pm 1.14\right)\cdot 10^{-3}\;{\mathrm{DN}/e}^{-} \end{array}$$

(7) $$\begin{array}{cc} K_{\mathrm{AB}} & =\left(6.14\pm 1.26\right)\cdot 10^{-3}\;{\mathrm{DN}/e}^{-}, \end{array}$$

for the cases with (AB) and without (noAB) anti-blooming scheme. The comparison of both values shows a slight difference, indicating that the conversion gain is hardly influenced by blooming. This result is not surprising since the variance depends only on the shot noise, respectively, the number of accumulated electrons $\mu_{e}$. Because this number is equal for both cases within the interval mentioned above, the conversion gains *K* are also equal for both cases.

This measurement shows that the conversion gain (or PTC measurement) in itself is not a reliable quantity to verify the proper function of a TDI sensor, since it does not reflect blooming effects.

#### 3.2.2. Signal-to-Noise Ratio

We have seen that the conversion gain is hardly influenced by blooming, whereas the light-dependent offset affects the responsivity gravely. To show how this offset affects further sensor characteristics, we will first examine the influence of blooming on the signal-to-noise ratio (SNR), which is given by:

(8) $$\begin{array}{c} \mathrm{SNR}\left(\mu_{p}\right)=\frac{K\eta \mu_{p}}{\sqrt{\sigma_{y.\mathrm{dark}}^{2}+K^{2}\eta \mu_{p}}} \end{array}$$

Here, η denotes the quantum efficiency that results from the fraction [15]

(9) $$\begin{array}{c} \eta =\frac{R}{K}. \end{array}$$

Since the responsivity *R* and the conversion gain *K* have been determined already, the SNR curves with and without the anti-blooming clocking mechanism can be calculated. Further, the SNR curve of an ideal sensor

(10) $$\begin{array}{c} {\mathrm{SNR}}_{\mathrm{ideal}}\left(\mu_{p}\right)=\sqrt{\mu_{p}}, \end{array}$$

is determined as well, where the quantum efficiency corresponds to η=1 and no dark noise $\sigma_{y.\mathrm{dark}}=0$ is present.

**Figure 9:**
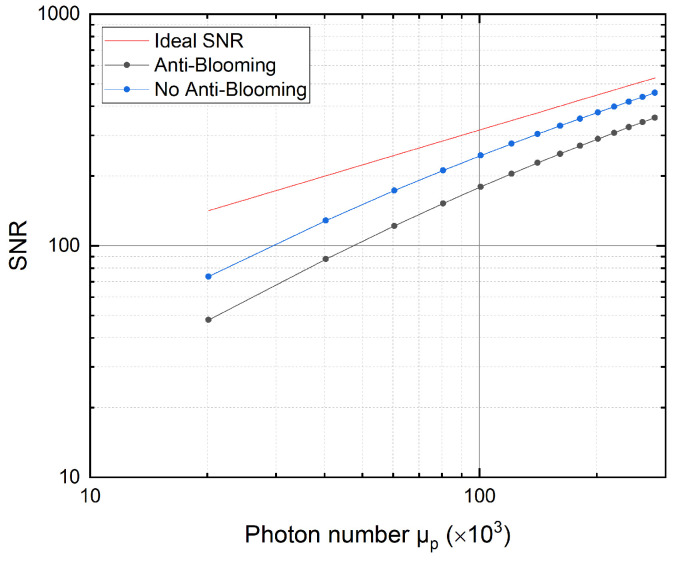
SNR of the sensor with and without anti-blooming clocking, as well as the SNR of an ideal sensor. Dots indicate the measured SNR curves, and the solid line corresponds to the ideal SNR of a sensor.

As shown in Figure 9, the measurement without anti-blooming clocking yields a better SNR curve than the measurement with anti-blooming clocking since it is closer to the ideal SNR curve. This is due to the dependence of the SNR on the quantum efficiency and, thus, the responsivity, as is shown in Equation (8).

Hence, because the responsivity contains a light-dependent offset as already mentioned in Section 3.1, the SNR also contains this light-dependent offset. Thus, the blooming of charge carriers of the unused stages distorts the actual SNR. As a consequence, further parameters, such as the dynamic range, will be affected, as shown in the following. The dynamic range is defined as the ratio

(11) $$\begin{array}{c} \mathrm{DR}=\frac{\mu_{p.\mathrm{sat}}}{\mu_{p.\min}}, \end{array}$$

where $\mu_{p.sat}$ is the photon number at the highest SNR, and $\mu_{p.min}$ is obtained for SNR=1. To calculate the photon numbers, we use the approximation [9]

(12) $$\begin{array}{c} \mu_{p}\left(\mathrm{SNR}\right)\approx \left\{\begin{array}{cc} \frac{{\mathrm{SNR}}^{2}}{\eta }\left(1+\frac{\sigma_{d}^{2}+\sigma_{q}^{2}/K^{2}}{{\mathrm{SNR}}^{2}}\right) & \mathrm{for}\;\mathrm{SNR}>>\sqrt{\sigma_{d}^{2}+\sigma_{q}^{2}/K^{2}}\\ \frac{\mathrm{SNR}}{\eta }\left(\sqrt{\sigma_{d}^{2}+\sigma_{q}^{2}/K^{2}}+\frac{\mathrm{SNR}}{2}\right) & \mathrm{for}\;\mathrm{SNR}<<\sqrt{\sigma_{d}^{2}+\sigma_{q}^{2}/K^{2}}. \end{array}\right. \end{array}$$

All determined parameters are listed in Table 1 for both cases to provide a better comparison.

As can be seen, the determined parameters differ in value for both mechanisms, since the measurement without anti-blooming is affected by overflowing charge carriers generated in unused stages. Consequently, the calculated values without anti-blooming are distorted considerably. To better understand how the parameters are distorted by blooming, one can consider the responsivity *R*, which is the input–output gain of a detector:

(13) $$\begin{array}{c} \mu_{y}-\mu_{y.\mathrm{dark}}=R\cdot \mu_{p} \end{array}$$

Calculating the ratio of both mechanisms measured for 64 TDI yields

(14) $$\begin{array}{c} \frac{{\left(\mu_{y}-\mu_{y.\mathrm{dark}}\right)}_{\mathrm{noAB}}}{{\left(\mu_{y}-\mu_{y.\mathrm{dark}}\right)}_{\mathrm{AB}}}=\frac{R_{\mathrm{noAB}}}{R_{\mathrm{AB}}}\cdot \frac{\mu_{p.\mathrm{noAB}}}{\mu_{p.\mathrm{AB}}}=1.4. \end{array}$$

However, the responsivity *R* is a constant and characteristic quantity for a detector [9], which should result in the same value independently of the number of TDI stages or used mechanisms. Therefore, it can be concluded that the input, i.e., the photon numbers differ according to

(15) $$\begin{array}{c} \mu_{p.\mathrm{noAB}}=1.4\cdot \mu_{p.\mathrm{AB}}. \end{array}$$

This difference can be explained as follows: If no anti-blooming mechanism is implemented, the photons generate charge carriers in the active and inactive regions of the CCD. Since the carriers in the inactive region are not removed, they start to overflow into the adjacent active area, contributing to the signal. Hence, the total signal is made up of charge carriers generated in the active pixel area A and partially of the charge carriers generated in the adjacent inactive pixel.

This can also be verified by comparing the quantum efficiencies of both implementations. In general, we would expect the same quantum efficiency, independently of the number of TDI stages, since it is defined as the probability that a photon of a particular wavelength generates an electron. With Equation (9), we obtain the measurement with anti-blooming the results

(16) $$\begin{array}{c} \eta_{128.\mathrm{AB}}=\eta_{64.\mathrm{AB}}=\eta_{32.\mathrm{AB}}=0.60 \end{array}$$

for the 128, 64, and 32 TDI stages. In contrast, we obtain the measurement without anti-blooming

(17) $$\begin{array}{c} \eta_{128.\mathrm{noAB}}=0.60,\;\;\;\;\;\;\;\;\;\;\;\eta_{64.\mathrm{noAB}}=0.8,\;\;\;\;\;\mathrm{and}\;\;\;\;\;\eta_{32.\mathrm{noAB}}=1.39. \end{array}$$

According to the definition, this is implausible, since these differences indicate a higher probability of generating electrons, i.e., a higher signal is obtained with the same number of photons if the number of active TDI stages is reduced. Hence, because the responsivity without anti-blooming is distorted, the responsivity-dependent quantities, such as the quantum efficiency, the signal-to-noise ratio, and the dynamic range, are distorted as well, yielding wrong results.

Consequently, before characterizing TDI sensors, the linearity of the signal in dependence on the TDI stage number has to be confirmed first to ensure that no undesired signal from the inactive region impacts the results. Otherwise, the measured sensor characteristics will strongly differ from the actual values of the sensor and degrade the image quality considerably if the linearity is not given.

Thus, the sensor parameters determined for the measurement without anti-blooming do not increase; they are distorted due to blooming and do not improve the sensor’s performance.

## 4. Discussion and Conclusions

In this paper, we have analyzed a TDI CCD that employs anti-blooming clocking. Measurements with and without this anti-blooming mechanism have been conducted and compared, showing considerable differences in the determined sensor parameters. These differences were caused due to blooming for the measurement without anti-blooming, contributing to the signal and resulting in responsivity curves that differ depending on the employed TDI stage number.

Nevertheless, since the responsivity is a constant parameter for a detector, the higher mean Gray values resulted from a higher number of photons. However, because blooming adds an unknown contribution to the actual signal generated in the active area, it makes the correct characterization of the sensor impossible and is, therefore, undesired. An anti-blooming mechanism was implemented to prevent this disadvantage by removing the charge carriers in the unused region by clocking them in the opposite direction.

Furthermore, since the quantum efficiency, the SNR, and the dynamic range depending on the responsivity, they have also been distorted by blooming, yielding wrong results and characteristics. This was verified by comparing the quantum efficiencies for both mechanisms. Here, the comparison of the parameters with deployed anti-blooming implementation resulted in equal responsivities and quantum efficiencies, independently of the number of used TDI stages, indicating the effective functioning of this mechanism.

Beyond that, blooming not only yields wrong sensor characteristics but also considerably degrades the image quality in terms of contrast since excess carriers are added to pixel signals yielding erroneous values.

In conclusion, it has been shown that using the anti-blooming clocking implementation led to accurate and correct measurements of the sensor characteristics. Consequently, TDI CCDs need to contain an anti-blooming mechanism. This can be accomplished by dumping the charge in a lateral or vertical anti-blooming drain or clocking them in the opposite direction into a drain, as this paper shows. Moreover, the linearity between different TDI stage modes can be used as a method to check the sensor’s functionality and to support the correctness of the measurements and sensor characterization. This is crucial, especially since many parameters used for characterization do not record the occurrence of blooming effects.

## Figures and Tables

**Table 1:** Determined parameters for measurements with and without an anti-blooming mechanism for 64 TDI.

Parameter	Anti-Blooming	No Anti-Blooming
Responsivity (DN)	$3.68\times 10^{-3}$	$5.14\times 10^{-3}$
Conversion Gain (DN/e${}^{-}$)	$6.14\times 10^{-3}$	$6.30\times 10^{-3}$
Quantum Efficiency	0.60	0.82
SNR	1–357	1–457
Dynamic Range	800	1270

## Data Availability

Not applicable.
